# Human cyclin T1 expression ameliorates a T-cell-specific transcriptional limitation for HIV in transgenic rats, but is not sufficient for a spreading infection of prototypic R5 HIV-1 strains *ex vivo*

**DOI:** 10.1186/1742-4690-6-2

**Published:** 2009-01-13

**Authors:** Nico Michel, Christine Goffinet, Kerstin Ganter, Ina Allespach, Vineet N KewalRamani, Mohammed Saifuddin, Dan R Littman, Warner C Greene, Mark A Goldsmith, Oliver T Keppler

**Affiliations:** 1 Department of Virology, University of Heidelberg, 69120 Heidelberg, Germany; 2 The Howard Hughes Medical Institute, Skirball Institute of Biomolecular Medicine, New York University School of Medicine, New York 10016, USA; 3 CONRAD, Eastern Virginia Medical School, 1911 North Fort Myer Drive, Suite 900, Arlington, Virginia 22209, USA; 4 Gladstone Institute of Virology and Immunology, and Departments of Medicine and Microbiology and Immunology, University of California, San Francisco, California 94158, USA; 5 Roche Diagnostics GmbH, Sandhoferstr. 116, 68305 Mannheim, Germany; 6 Department of Microbiology and Molecular Genetics, Medical College of Winsconsin, 8701 Watertown Plank Road, Milwaukee, Wisconsin, USA; 7 Cogentus Pharmaceuticals, Menlo Park, California, USA

## Abstract

**Background:**

Cells derived from native rodents have limits at distinct steps of HIV replication. Rat primary CD4 T-cells, but not macrophages, display a profound transcriptional deficit that is ameliorated by transient trans-complementation with the human Tat-interacting protein Cyclin T1 (hCycT1).

**Results:**

Here, we generated transgenic rats that selectively express hCycT1 in CD4 T-cells and macrophages. hCycT1 expression in rat T-cells boosted early HIV gene expression to levels approaching those in infected primary human T-cells. hCycT1 expression was necessary, but not sufficient, to enhance HIV transcription in T-cells from individual transgenic animals, indicating that endogenous cellular factors are critical co-regulators of HIV gene expression in rats. T-cells from hCD4/hCCR5/hCycT1-transgenic rats did not support productive infection of prototypic wild-type R5 HIV-1 strains *ex vivo*, suggesting one or more significant limitation in the late phase of the replication cycle in this primary rodent cell type. Remarkably, we identify a replication-competent HIV-1 GFP reporter strain (R7/3 YU-2 Env) that displays characteristics of a spreading, primarily cell-to-cell-mediated infection in primary T-cells from hCD4/hCCR5-transgenic rats. Moreover, the replication of this recombinant HIV-1 strain was significantly enhanced by hCycT1 transgenesis. The viral determinants of this so far unique replicative ability are currently unknown.

**Conclusion:**

Thus, hCycT1 expression is beneficial to *de novo *HIV infection in a transgenic rat model, but additional genetic manipulations of the host or virus are required to achieve full permissivity.

## Background

*In vivo *studies on HIV-1 pathogenesis and the testing of antiviral strategies have been hampered by the lack of an immunocompetent small animal that is fully permissive for infection. The host range and cell tropism of HIV-1 is highly restricted: it can only efficiently replicate in primary and immortalized T-cells and macrophages of human origin. Cells from rats and mice do not or only inefficiently support various steps of the HIV-1 replication cycle [1-6]. Molecular characterization of some of these species-specific barriers has revealed the inability of several rodent orthologues of cellular factors, essential for HIV replication in human cells, to support distinct viral functions. The entry of HIV-1 provides a compelling example: the CD4 binding receptor and the chemokine co-receptors CCR5 or CXCR4 from rodents generally cannot support viral entry [1,7-10]. Expression of the human HIV-1 receptor complex largely overcomes the entry restriction, and this observation has spured efforts to develop transgenic (-tg) mouse and rat models permissive for HIV replication through a block-by-block humanization (for an overview [11]). This conceptual approach seeks to surmount intrinsic limitations in the HIV-1 replication cycle in small animals by stable introduction of critical human transgenes into the genome of laboratory rodents using transgene or knock-in technology.

Consequently, we generated Sprague-Dawley rats that transgenically express hCD4 and hCCR5 selectively on CD4 T-cells, macrophages, and microglia [12], the major targets for productive HIV-1 infection in humans. After a systemic challenge with HIV-1_YU-2_, these double-tg animals harboured significant levels of HIV-1 cDNAs in lymphatic organs [7,12,13], up to 10^6 ^HIV-1 cDNA copies per 10^6 ^splenocytes [7], demonstrating a robust susceptibility to HIV-1 *in vivo*. This level of susceptibility was several orders of magnitude higher than in comparable tg mouse or rabbit models [2,5,14] and allowed a preclinical proof-of-principle efficacy study for a peptidic HIV entry inhibitor and a reverse transcriptase inhibitor [7].

Despite this advancement, significant limitations exist in the current model: levels of plasma viremia are low and only transient [12]. To a large extent, these limitations may be due to a cell type-specific block to productive HIV-1 infection in hCD4/hCCR5-tg rats. Primary T-cells, in contrast to macrophages from these animals, did not support a productive R5 HIV-1 infection [7,12]. Following up on this observation, we recently compared the efficiency of early steps of the HIV replication cycle in infected primary T-cells from hCD4/hCCR5-tg rats and human donors. Remarkably, levels of viral entry, HIV-1 cDNA production, nuclear import of the preintegration complex, as well as the frequency of integration into the host genome, were similar in both species [3]. In contrast, a profound post-entry impairment was evident for early HIV gene expression in primary rat T-cells [3]. We reasoned that a transcriptional deficit due to an inefficient Tat-dependent HIV-1 LTR transactivation may underlie this inefficient viral gene expression in rats as it does in mice [15,16]. Cyclin T1 (CycT1) is a key component of the positive transcription elongation factor b (P-TEFb) [6], which is critical for efficient elongation of many cellular as well as HIV transcripts (for review [17]). In mice, the inability of CycT1 to support the interaction with the transactivation response (TAR) element when bound to Tat has been mapped to one critical amino acid (tyrosine-261; cytosine-261 in hCycT1) [18-21]. Intriguingly, rat and mouse CycT1 have a 96% sequence homology and both contain tyrosine-261 [4]. While ectopic expression of hCycT1 in NIH3T3 cells resulted in a marked, ~10- to 100-fold enhancement of LTR-driven gene expression, this effect was quite moderate, only ~3-fold in Rat2 cells [1,4,6,9], challenging the potential benefit of ectopic expression of hCycT1 in the rat species. However, evidence in support of such an approach was provided by an experiment, in which transient coexpression of hCycT1 and proviral HIV reporter DNA in nucleofected primary rat T-cells resulted in a marked enhancement of early viral gene expression [3]. This suggested that an underlying transcriptional defect linked to the non-functional rat orthologue was, at least in part, responsible for the gene expression phenotype in native rat T-cells.

In NIH3T3 or Rat2 cells, additional less-defined blocks in the late phase of the HIV-1 replication cycle add up to a profound drop in the yield of viral progeny, up to 10^4^-fold or 10^2^-fold, respectively, from a single round of replication [1,4,9,12,22,23]. In both the mouse and rat fibroblast cell line, these late-stage barriers display a recessive phenotype and likely result from non-functional rodent cofactors since they can be surmounted in rodent-human heterokaryons. In striking contrast to all mouse cell line studies, mice that carry a full-length HIV-1 provirus have been reported to secrete high levels of infectious HIV-1 with viremia levels of >60,000 HIV RNA copies per ml [24]. Moreover, in T-cells and macrophages from these provirus-carrying mice, tg co-expression of hCycT1 markedly boosted HIV-1 transcription and virus production [25,26].

On a more general level, the transcriptional phenotype as well as the severe late-phase limitations described in rodent cell lines may thus not necessarily be predictive for the ability of primary cells to support these steps of the HIV-1 replication cycle. In the current study, we generated rats that transgenically express hCycT1 in a cell type-specific manner to explore their suitability for enhancing HIV-1 transcription and gene expression in primary T-cells and macrophages. Moreover, we wanted to probe whether ameliorating the transcriptional deficit by hCycT1 transgenesis may render primary T-cells from rats that transgenically co-express the HIV receptor complex susceptible for a productive and spreading R5 HIV-1 infection.

## Results

### Construction of tg rats that selectively express hCycT1 in HIV target cells

**Figure 1:**
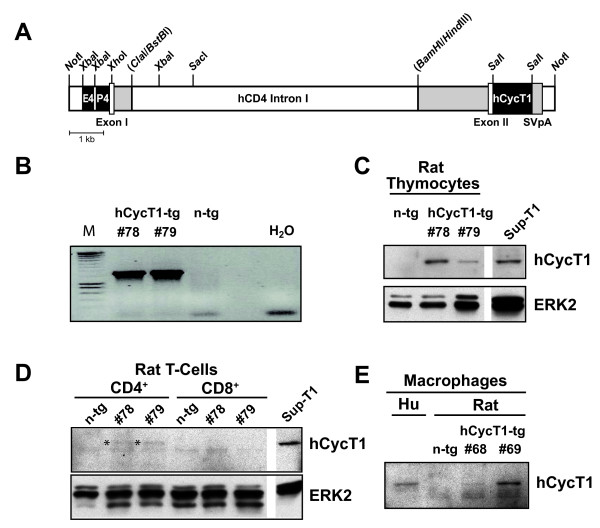
**Expression of hCycT1 in CD4 T-cells and macrophages from hCycT1-tg rats**. (A) Schematic representation of the tg vector for hCycT1, pMΦE4A.CyclinT1. (For details, see "Methods" and [25]. E4/P4: murine *CD4 *enhancer/promoter. (B) hCycT1 transgene-specific PCR amplifying a diagnostic 1.7-kb fragment in tail DNA from hCycT1-tg rats. M: DNA marker. (C-E) Western blot analysis of hCycT1 expression in extracts from (C) thymocytes, (D) spleen-derived CD4 T-cells and CD8 T-cells, enriched by magnetic bead selection, or (E) spleen-derived macrophages, using an antibody specific for CycT1 of human origin. Cells from n-tg littermates, or human Sup-T1 T-cells and human MDMs served as negative and positive controls, respectively. (C, D) Blots were reprobed for ERK2 as loading reference. (D) * indicates the hCycT1-specific band. The lower band seen in all splenocyte samples was considered non-specific. # gives the ID numbers of individual tg rats.

To selectively express hCycT1 in the most relevant HIV-1 target cells, we employed a chimeric mouse/human transgene vector (Fig. 1A) that directs expression of cDNA inserts exclusively in CD4 T-cells and cells from the monocyte/macrophage lineage. This strategy has been employed to generate hCCR5-tg rats [12] as well as hCXCR4-tg rats (O.T.K. and M.A.G., unpublished). Several independent rat lines tg for hCycT1 were developed by pronuclear microinjection of fertilized oocytes from outbred Sprague-Dawley rats. Five hCycT1 integration founders were identified by a transgene-specific PCR, which amplifies a ~1.7-kb fragment (Fig. 1B), and four of these founders transmitted the transgene to their progeny (data not shown).

All four tg rat lines expressed significant levels of hCycT1 in thymocyte extracts as assessed by a species-specific western blot, and founder line 44, displaying the highest hCycT1 level, was selected for further studies (data not shown). F2 progeny did not reveal any gross histopathology (data not shown), and offspring from this hCycT1-tg line have generally been healthy. The expression pattern of hCycT1 was examined in select tissues and purified cell populations from hCycT1-tg rats (Fig. 1C–E). First, hCycT1 expression was readily detectable in rCD4 T-cell-rich thymocyte extracts from hCycT1-tg rats, but not from a non-tg (n-tg) littermate (Fig. 1C). Second, the T-cell subset-specific expression of hCycT1 was analyzed in rCD4- and rCD8-positive splenocytes separated by antibody-coupled magnetic beads (purities of 94% and 93%, respectively; data not shown). A low, but significant hCycT1 expression was detectable only in the rCD4-positive, but not in the rCD8-positive, purified splenocyte fractions of both hCycT1-tg animals (Fig. 1D, * hCycT1). Third, hCycT1 expression was found in spleen-derived macrophages from the two hCycT1-tg rats tested as well as monocyte-derived macrophages (MDM) from a human donor, but not in macrophages from a n-tg control rat (Fig. 1E). Thus, expression of hCycT1 has been targeted to the desired, biologically relevant cell types in tg rats. This finding is consistent with the exclusive expression of hCCR5 or hCD4 on these rat cells, employing the identical or a closely related transgene vector backbone, respectively [12], and with the targeted expression of hCycT1 in tg mice [10,25,26]. Furthermore, it provides the conceptual basis to generate potentially more susceptible rats through interbreeding of these different tg rat lines to achieve expression of all of these human transgenes in the same HIV target cells.

### Primary T-cells from hCycT1-tg rats support markedly elevated levels of early HIV gene expression

**Figure 2:**
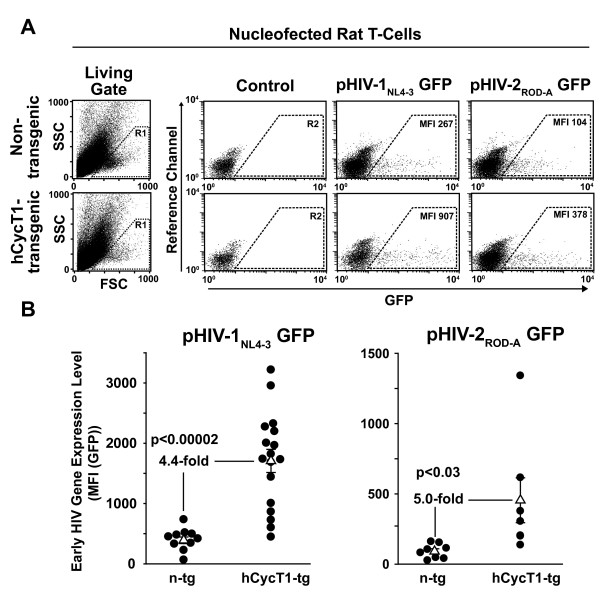
**Enhanced early HIV-1_NL4-3 _and HIV-2_ROD-A _gene expression in primary T-cells from hCycT1-tg rats after nucleofection of proviral DNA**. Activated T-cells from hCycT1-tg and n-tg rats were nucleofected with proviral GFP reporter constructs pHIV-1_NL4-3 _GFP or pHIV-2_ROD-A _GFP in principle as reported [3,27]. The GFP expression level in viable cells was analyzed 24 h later by flow cytometry. (A) Representative flow cytometry dot plots of nucleofected T-cells. Living cells were identified by their forward scatter (FSC) and side scatter (SSC) characteristics (left panels, R1 gate). The GFP fluorescence in living cells was analyzed against an empty reference channel (right panels, FL-4). The MFI of GFP-expressing cells (right panels, R2 gate) was determined as a surrogate marker for early viral gene expression. (B) Cumulative results from several independent experiments. Each closed circle depicts the MFI of provirus-nucleofected T-cells as the mean of triplicates performed for cultures from individual animals. Open triangles represent the arithmetic mean of the MFI of all animals in one group ± SEM. The indicated *p*-values were calculated using the unpaired Student's *t*-test.

As a first functional characterization, activated T-cells from hCycT1-tg rats and n-tg littermates were transfected with proviral GFP reporter plasmids, pHIV-1_NL4-3 _GFP or pHIV-2_ROD-A _GFP, with a species-adapted nucleofection protocol [27], and analyzed for GFP expression in viable cells one day later (Fig. 2A). In these proviral reporter constructs, GFP is expressed in a Rev-independent manner from the *nef *locus. hCycT1 transgenesis resulted in an average enhancement of early HIV gene expression, as measured by the GFP mean fluorescence intensity (MFI) of nucleofected cells, of 4.4-fold for HIV-1 (p < 0.00002; unpaired Student's *t*-test; Fig. 2B, left panel) and of 5-fold for HIV-2 (p < 0.03; Fig. 2B, right panel).

**Figure 3:**
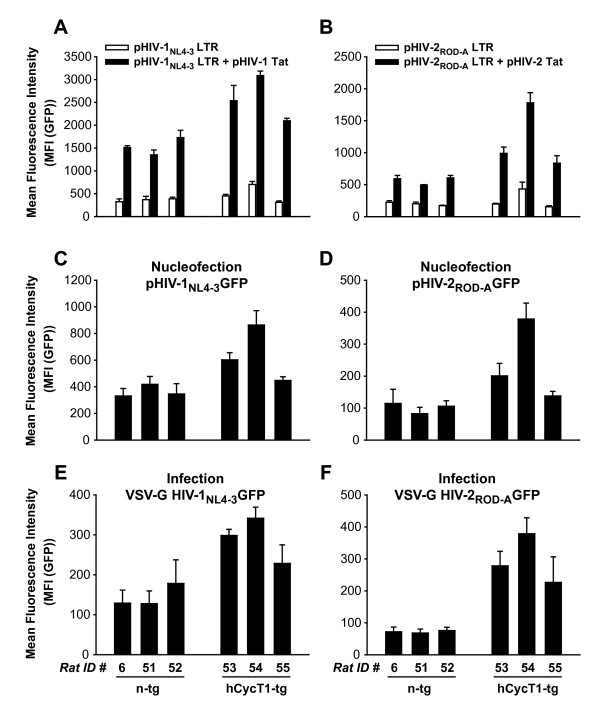
**hCycT1 transgenensis enhances the Tat-responsiveness of rat T-cells**. Activated, spleen-derived T-cells from three hCycT1-tg and three n-tg rats were nucleofected with (A, B) minimal reporter constructs pHIV-1_NL4-3 _LTR GFP or pHIV-2_ROD-A _LTR GFP in the presence or absence of HIV-1 Tat or HIV-2 Tat expression constructs, respectively, or (C, D) full-length proviral GFP reporter constructs pHIV-1_NL4-3 _GFP or pHIV-2_ROD-A_. Analysis of gene expression was performed as in Fig. 2. (E, F) In addition, T-cell cultures from the same rats were infected with corresponding VSV-G pseudotyped HIV-1_NL4-3 _GFP or HIV-2_ROD-A _GFP viruses and analyzed for reporter gene expression by flow cytometry three days later. Given are the arithmetic mean + SD of triplicates.

To dissect the contribution of Tat-dependent and Tat-independent LTR-driven transcription for the enhancement of early viral gene expression mediated by hCycT1 transgenesis in rat T-cells, we constructed minimal reporter plasmids consisting of the complete, PCR-amplified LTR and Gag-leader sequences from either HIV-1_NL4-3 _or pHIV-2_ROD-A_, which drive the expression of GFP (pHIV LTR GFP). Activated T-cells from 3 n-tg and 3 hCycT1-tg rats were nucleofected with either pHIV-1_NL4-3 _LTR GFP or pHIV-2_ROD-A _LTR GFP in the presence or absence of expression plasmids encoding for HIV-1 Tat and HIV-2 Tat, respectively, and analyzed by flow cytometry one day later. The basal, Tat-independent LTR activity was comparable for both groups of animals irrespective of the hCycT1 transgene status (Fig. 3A, B; open histograms). Importantly, co-expression of Tat elevated levels of early gene expression in T-cells from the group of n-tg rats by 4-fold (HIV-1) and 3-fold (HIV-2) and, notably, 6-fold (HIV-1) and 5-fold (HIV-2) in hCycT1-tg T-cells (Fig. 3A, B; filled histograms). Furthermore, parallel nucleofection studies of T-cell cultures from the identical animals with the corresponding full-length proviral constructs showed a ~2-fold enhancement in this limited set of animals (Fig. 3C, D). Moreover, this phenotype was largely recapitulated in single-round infection experiments with VSV-G pseudotyped stocks of these HIV strains, assessing GFP expression on day 3 after infection (2-fold for HIV-1 and 4-fold for HIV-2; Fig. 3E, F).

**Figure 4:**
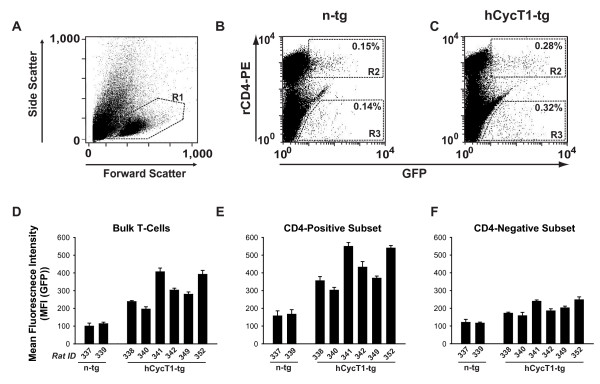
**Functional analysis of hCycT1 transgenesis in T-cell bulk cultures from rats mirrors the subset-specific impact in CD4 T-cells**. Activated, spleen-derived T-cell cultures from six hCycT1-tg and two n-tg rats were infected with VSV-G HIV-1_NL4-3 _GFP. (A-C) Three days later, cells were stained with a rCD4-PE antibody (clone OX-8) and analyzed for GFP expression in CD4-positive or CD4-negative T-cells. Shown are representative flow cytometry dot plots of (A) viable cells identified by FSC/SSC characteristics (R1 gate), or infected, stained T-cells from one (B) n-tg or (C) hCycT1-tg rat with double-positive cells and corresponding MFI values indicated in gates R2 (rCD4-positive cells) and R3 (rCD4-negative cells). The percentage of rCD4-positive cells of all viable, rCD3-positive lymphocytes were (B) 61% (and 39% CD8-positive T-cells), and (C) 27% (and 73% CD8-positive T-cells). The CD4low sub-population in B and C likely reflects residual monocytes. (D-E) Quantitative analysis of early HIV-1 gene expression, represented by the MFI (GFP) in infected T-cell subsets: (D) bulk T-cells, (E) CD4-positive T-cells, (F) CD4-negative T-cells. Histogram bars depict the arithmetic means + SD of triplicates.

These mitogen/IL-2 activated rat splenocyte cultures are comprised of both CD4- and CD8-positive T-cells. Antibody-coupled magnetic bead enrichment of CD4 T-cells, unfortunately, interferes with their viability, proliferative capacity, and subsequent HIV susceptibility (data not shown), and could thus not be used for functional analyses of T-cells from transgenic animals. We thus investigated in more detail the consequences of VSV-G pseudotyped HIV-1 GFP infection of these splenocyte-derived T-cell bulk cultures. First, the relative percentage of CD4 T-cells was independent of the transgene status and quite variable ranging from 6 to 72% (Fig. 4B, C, and data not shown). Importantly, the hCycT1-mediated enhancement of early HIV-1 gene expression seen in the analysis of infected T-cell bulk cultures (Fig. 4D), closely matched the enhancement of gene expression in the subset of CD4 T-cells (Fig. 4E) on the level of individual animals. Of note, also a slight enhancing effect was observed in the CD4-negative population (Fig. 4F), possibly reflecting a leakage of transgene expression into the CD8 T-cells subset, despite exclusive detection of hCycT1 in CD4 T-cells (Fig. 1D). Overall, the degree of hCycT1-mediated enhancement of early gene expression was slightly less pronounced in the bulk cultures compared to the CD4 T-cells (compare Figs. 4D and 4E). Thus, the analysis of HIV gene expression in VSV-G HIV-1 pseudotype-infected bulk cultures of activated rat splenocytes in the context of hCycT1 transgenesis reflects to a large degree the situation in the CD4 T-cell subset.

**Figure 5:**
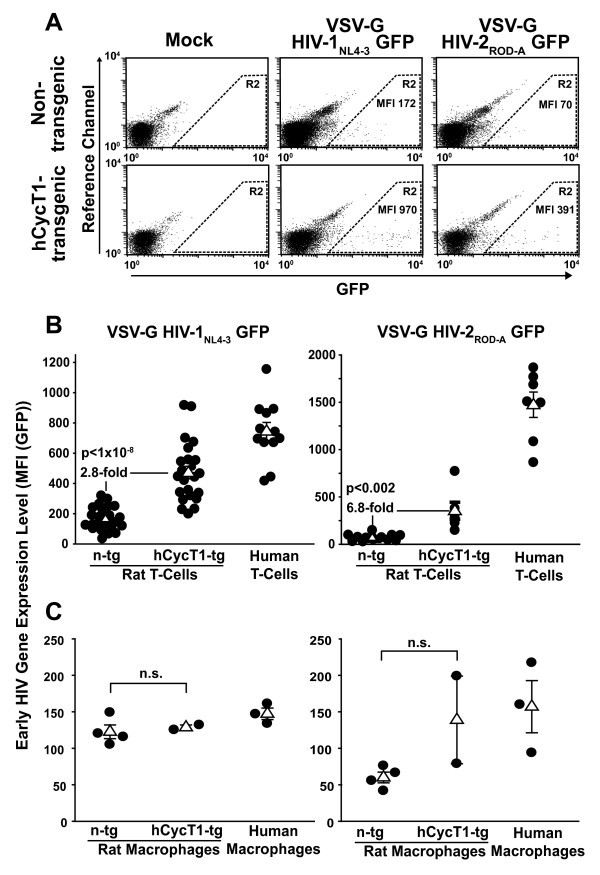
**hCycT1 transgenesis boosts early HIV gene expression in infected primary T-cells, but not in macrophages**. (A, B)Activated T-cells from hCycT1-tg and n-tg rats were infected with VSV-G HIV-1_NL4-3 _or HIV-2_ROD-A _GFP reporter viruses and analyzed by flow cytometry three days later. (B, C) Cumulative results from several independent infection experiments of cells derived from rats or human donors. Closed circles depict the MFI of (B) HIV-infected T-cells or (C) HIV-infected macrophages as the mean of triplicates of experiments performed for cultures from individual donors. Open triangles represent the arithmetic mean of the MFI of all donors in one group ± SEM. n.s. = not significant.

Furthermore, examination of infected bulk T-cell cultures from a large cohort of animals corroborated that hCycT1-tg rats displayed significantly higher HIV gene expression than n-tg controls, on average 2.8-fold for HIV-1_NL4-3 _(p < 1 × 10^-8^) and 6.9-fold for HIV-2_ROD-A _(p < 0.002) (Fig. 5A, B). The percentage of infected, GFP-positive cells ranged from 0.2-2% (data not shown). Of note, absolute levels and hCycT1-dependent enhancement of early HIV-1 gene expression were nearly identical in rat splenocyte cultures activated by either IL-2 alone or IL-7 alone compared to the cultures activated by the standard protocol using ConA/IL-2 (data not shown). With infected T-cells from human donors providing a critical reference, hCycT1 transgenesis markedly narrowed the rat-human species gap for early HIV-1_NL4-3 _gene expression from a difference of 4.5-fold for n-tg rats down to 1.6-fold for hCycT1-tg rats (Fig. 5B, left panel). For HIV-2_ROD-A_, this gap narrowed from 32.9-fold for n-tg rat T-cells down to 4.8-fold for hCycT1-tg T-cells (Fig. 5B, right panel). Remarkably, infected T-cells from ~1/4 of hCycT1-tg rats supported early HIV-1_NL4-3 _gene expression at levels within the average range of infected human T-cells (Fig. 5B, left panel). Of note, considerable heterogeneity in levels of early HIV gene expression supported by T-cells derived from individual hCycT1-tg animals as well as from individual human donors was observed both in provirus nucleofection and HIV infection studies (Fig. 2B, 4D, 5B; and data not shown).

We expanded our HIV gene expression analysis to rat macrophages, the second major HIV target population. As reported, early HIV-1_NL4-3 _gene expression was comparable and statistically indistinguishable for infected macrophages derived from n-tg rats and MDM from human donors [3]. Here, hCycT1 transgenesis did not have an enhancing effect (Fig. 5C, left panel). For HIV-2_ROD-A_, a trend towards higher viral gene expression was observed for macrophages from hCycT1-tg rats compared to n-tg rats, but this difference did not reach statistical significance (Fig. 5C, right panel). Thus, infected primary macrophages from rats display levels of early HIV gene expression similar to those in human MDM, indicating that hCycT1 transgenesis is not a requirement for robust HIV gene expression in the monocyte/macrophage lineage in rats. Collectively, these results show a cell type-specific ability of primary rat cells to support HIV LTR-driven early gene expression and to allow an elevation of early HIV gene expression upon tg expression of hCycT1. The elevated levels of gene expression from the HIV *nef *locus reach human levels in infected T-cells from some hCycT1-tg rats.

### HIV gene expression in transgenic rat T-cells

**Figure 6:**
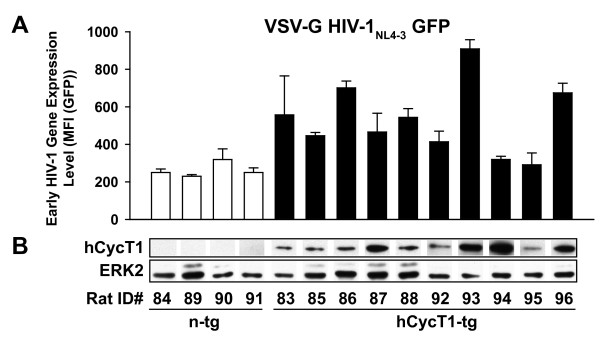
**Early HIV-1_NL4-3 _gene expression in infected T-cells from individual hCycT1-tg rats is variable, and the degree of enhancement does not correlate with hCycT1 steady-state levels**. (A) T-cells derived from 10 hCycT1-tg and four n-tg rats were infected with VSV-G HIV-1_NL4-3 _GFP and analyzed three days later as described in the legend to Fig. 3. Levels of CD4 T-cells in these cultures ranged from 7 to 26% (and 74 to 93% CD8 T-cells). The MFI of all GFP-expressing, infected cells was determined, and the arithmetic mean ± SD of triplicates is given for one experiment. At the time of HIV-1 infection, uninfected T-cells were harvested for (B) western blot analysis to assess the expression of hCycT1, with ERK2 serving as a loading control.

In light of the heterogeneity in HIV gene expression in T-cells from hCycT1-tg rats, but not from n-tg rats (Figs. 2B, 5B), we wondered whether expression levels of the human transgene may be rate-limiting. In activated, infected T-cells, we therefore explored the relationship between the expression levels of hCycT1 and the ability to support early viral gene expression. Three days after infection with VSV-G HIV-1_NL4-3 _GFP, cultures derived from 10 heterozygous hCycT1-tg rats supported early HIV-1 gene expression at quite variable levels (Fig. 6A, filled bars). Accordingly, these rat T-cell cultures were grouped into three phenotypic responder categories: low (rat ID 94, 95; MFI < 320), intermediate (rat ID 83, 85, 87, 88, 92; MFI = 400-600), and high responders (rat ID 86, 93, 96; MFI > 600). Cultures derived from the four n-tg animals supported only low levels of viral gene expression levels (Fig. 6A, open bars; MFI<320), and the results for which were statistically indistinguishable from the low-responder group among the hCycT1-tg animals. At the time of infection, a cell aliquot was harvested to determine levels of hCycT1 expression in lysates by species-specific western blot analysis. The steady-state expression level of hCycT1 differed markedly between cultures from individual rats (Fig. 6B); however, no correlation with the phenotype of infected cultures for early HIV gene expression (Fig. 6A) could be established. For example, cultures from individual tg rats with very high hCycT1 expression levels (rat ID 93, 94) expressed either high (93) or low (94) HIV GFP reporter levels upon infection.

This transcriptional phenotype was found to be a stable characteristic of individual rats: comparable results were obtained for several independently established T-cell cultures from blood draws of the same tg animals on different days (data not shown). These results indicate that tg expression of hCycT1 is necessary for enhancing early HIV gene expression in infected primary rat T-cells, but currently unknown endogenous factors appear to play an additional critical regulatory role for HIV gene expression.

### T-cells from triple-tg rats do not support a productive HIV-1 infection, despite enhanced early gene expression

Previously, we found that T-cells from hCD4/hCCR5-tg rats do not support a spreading HIV-1 infection, despite efficient virion entry [3,7,12]. Here, we determined if the enhancement in viral gene expression mediated by hCycT1 transgenesis could translate into a beneficial effect in the context of an infection with replication-competent R5 HIV-1 viruses. Triple-tg rats, heterozygous for hCD4, hCCR5 and hCycT1, were obtained through interbreeding, and their transgene status was determined by flow cytometry (hCD4/hCCR5) and PCR (hCycT1).

**Figure 7:**
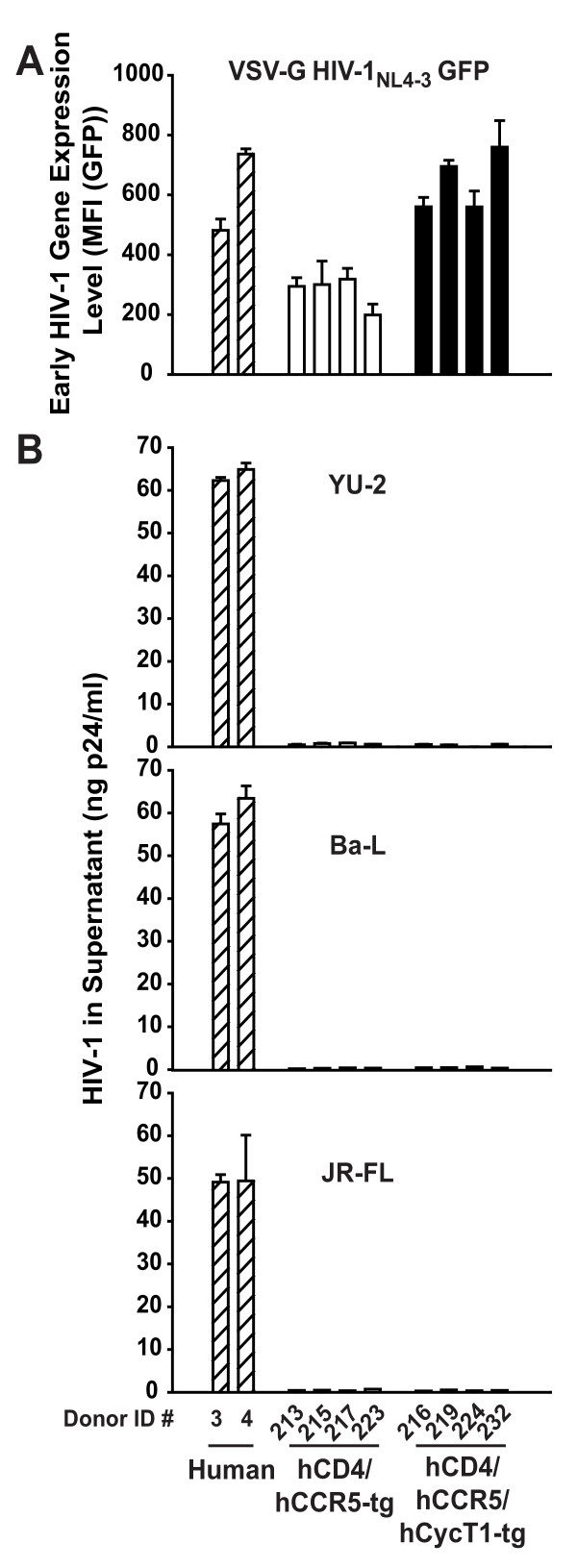
**T-cells from hCD4/hCCR5/hCycT1-triple-tg rats do not release virions into the culture supernatant following infection by several R5 HIV-1 strains, despite enhanced transcriptional activity**. Activated T-cells derived from the spleens of four double- or four triple-tg rats, or from PBMC of two human donors were plated in 96-well round bottom plates and were challenged with (A) single-round VSV-G HIV-1_NL4-3 _GFP reporter virus and on day 3 p.i., the MFI (GFP) was quantified by flow cytometry as a surrogate for early HIV-1 gene expression. (B) In parallel, T-cells were challenged with replication-competent R5 HIV-1 strains YU-2, Ba-L, or JR-FL (each 5 ng p24 per 2 × 10^6 ^cells) overnight. Cultures were washed twice with PBS and continuously cultivated for 2 weeks. At day 7 and 13 p.i., supernatant aliquots were removed and analyzed for the p24 concentration by antigen ELISA. Histograms depict the arithmetic mean ± SD of triplicates of values on day 13 p.i. from one experiment.

Spleen-derived T-cell cultures from four such animals were first characterized for their transcriptional phenotype after VSV-G HIV-1_NL4-3 _GFP infection. Spleen-derived T-cell cultures from four hCD4/hCCR5-tg littermates and activated PBMCs from two human donors served as references. All triple-tg cultures displayed enhanced early HIV-1 gene expression relative to double-tg controls (on average ~2.3-fold) and reached levels comparable to T-cells from the human donors (Fig. 7A). In parallel, T-cells were challenged with the R5 HIV-1 strains YU-2, Ba-L, or JR-FL (corresponding to either 5 or 50 ng p24 per 2 × 10^6 ^cells) and washed extensively the following day. Productive infection was followed by the concentration of p24 antigen in culture supernatants. Neither on day 7 p.i. (data not shown) nor on day 13 p.i. (Fig. 7B and data not shown) could significant p24 levels be detected in supernatants from any of the double- or triple-tg rat cultures. Expectedly, human reference T-cell cultures supported a productive, spreading, and efavirenz-sensitive infection for all of these HIV-1 strains (Fig. 7B and data not shown). Thus, despite largely overcoming the transcriptional deficit through hCycT1 expression, T-cells from triple-tg rats do apparently not support a productive HIV-1 infection.

### Identification of an HIV-1 GFP reporter virus that displays characteristics of a spreading infection in primary T-cells from hCD4/hCCR5-tg rats

**Figure 8:**
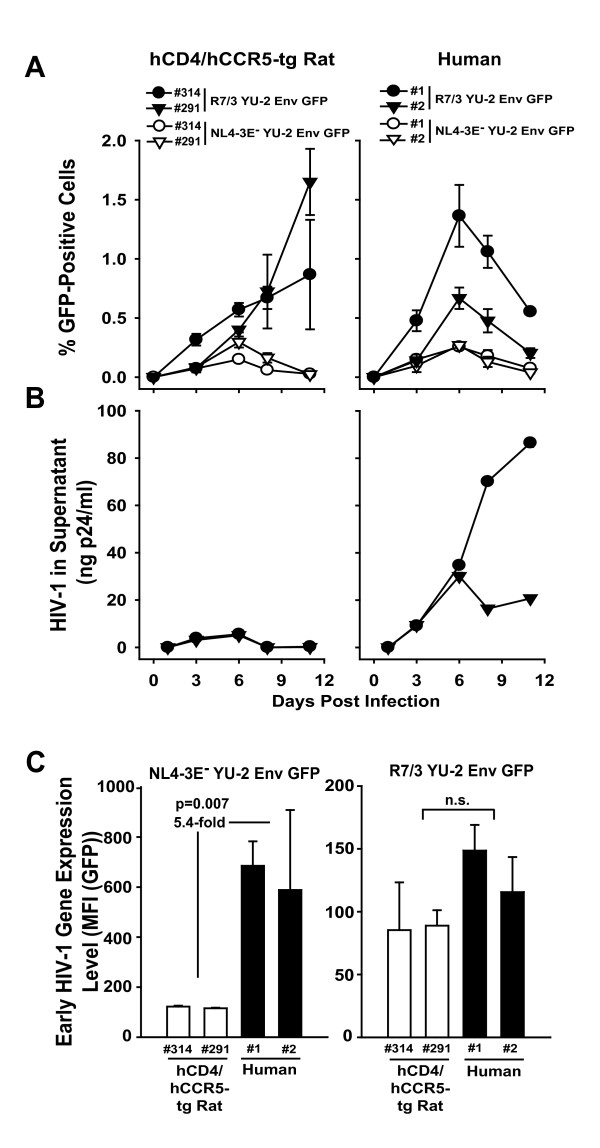
**Evidence for a spreading infection of a recombinant HIV-1 GFP reporter virus in rat T-cells carrying the HIV receptor complex**. Activated T-cells derived from two hCD4/hCCR5-tg rats and two human donors were challenged with either replication-competent HIV-1_R7/3 _YU-2 Env GFP virus or single-round YU-2 Env pseudotyped HIV-1_NL4-3 _E^- ^GFP virus (each 500 ng p24 per 2 × 10^6 ^cells) and analyzed at the indicated time points p.i. for (A) percentage of GFP-positive cells by flow cytometry and (B) the p24 concentration in culture supernatants by antigen ELISA until day 12 p.i. (C) The data obtained on day 3 under (A) were also analyzed for the MFI of the GFP-expressing cells as a surrogate for early HIV-1 gene expression, analogous to the procedure described in the legend to Fig. 2. Data shown are arithmetic means ± SD of triplicates of one experiment, which is representative for two to five independent experiments. For the data in (C) a Student's *t*-test was performed and significance is indicated. n.s. = not significant.

Besides the above reported R5 HIV-1 wildtype strains, we tested a replication-competent R5 HIV-1 reporter virus, R7/3 YU-2 Env GFP, which is a derivative of the T-cell tropic HIV-1_HXB2d _isolate that carries the *env *gene of HIV-1_YU-2 _and an *egfp *gene inserted into the *nef *locus [12,28]. This virus allows a sensitive and kinetic analysis of infected, GFP-expressing cells by flow cytometry and thus also provides an additional virological readout of infection besides p24 antigen levels in culture supernatants. Peripheral blood-derived T-cell cultures from hCD4/hCCR5-tg rats were challenged with HIV-1_R7/3 _YU-2 Env GFP overnight, washed, and continuously cultivated for up to two weeks. We observed a marked increase of the percentage of GFP-expressing CD4 T-cells from day 3 until day 10 p.i.. Peak levels of infected, GFP-expressing cells were comparable to those observed in infected human T-cells analyzed in parallel, while the latter typically showed a faster kinetic (Fig. 8A and data not shown). Parallel sampling of culture supernatants and p24 quantification, however, revealed a key discrepancy of infection characteristics between both species: while human T-cell cultures showed increasing levels of viral capsid antigen in the supernatant over the course of the experiment, the p24 concentrations in rat supernatants remained at background levels (Fig. 8B). Consistent with this finding, transfer of cell-free supernatants from HIV-1_R7/3 _YU-2 Env GFP-infected double-tg rat T-cells onto naïve rat cultures did not initiate an infection, while transfer of infected hCD4/hCCR5-tg T-cells again led to a steady increase of GFP-positive rat CD4 T-cells in the recipient culture (data not shown).

We also explored whether HIV-1_R7/3 _YU-2 Env GFP differs in its transcriptional phenotype in the rat-human species comparison from HIV-1_NL4-3 _GFP, the latter being the HIV-1 strain used in the experiments described above (Figs. 2, 3, 4, 5, 6, 7). Interestingly, analysis of the MFI of GFP of the infected T-cells as a surrogate for levels of early HIV-1 gene expression revealed that HIV-1_R7/3_YU-2 Env GFP did not display a significant difference between the two species (Fig. 8C, right panel). In contrast, HIV-1_NL4-3 _GFP showed a marked, on average 5.4-fold reduction (p = 0.007) in T-cells from these n-tg rat donors compared to the human reference controls (Fig. 8C, left panel), confirming the above described transcriptional phenotype for this viral strain (Figs. 2, 3, 4, 5, 6, 7). Thus, the HIV transcriptional phenotype in rat cells markedly depends on the employed HIV-1 strain and HIV-1_R7/3 _YU-2 Env GFP is superior to the HIV-1_NL4-3_-based virus.

**Figure 9:**
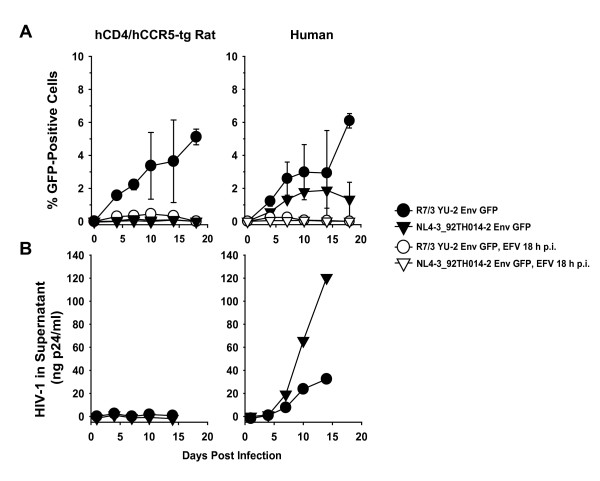
**The spread of HIV-1_R7/3 _YU-2 Env GFP in hCD4/hCCR5-tg rat T-cells is sensitive to a reverse transcriptase inhibitor and not seen for a second replication-competent R5 HIV-1 GFP reporter virus**. Activated T-cells derived from a hCD4/hCCR5-tg rat and a human donor were challenged with either replication-competent HIV-1_R7/3 _YU-2 Env GFP virus or replication-competent HIV-1_NL4-3 _92TH014-2 Env GFP virus (each 500 ng p24 per 2 × 10^6 ^cells). 18 h p.i. the infected wells were split and cultivation was continued in the presence or absence of efavirenz (EFV) (5 μM). At the indicated time points, the infection was analyzed for (A) the percentage of GFP-positive cells by flow cytometry and (B) the p24 concentration in culture supernatants by antigen ELISA. Data shown are arithmetic means ± SD of triplicates.

The steady rise of the percentage of GFP-expressing rat CD4 T-cells over the course of the HIV-1_R7/3 _YU-2 Env GFP infection (Fig. 9A) was sensitive to the addition of the reverse transcriptase inhibitor efavirenz (EFV) 18 h post-challenge, which inhibited viral replication subsequent to first-round infection. Similarly, the elevation of the percentage of GFP-positive T-cells over time following HIV-1_R7/3_YU-2 Env GFP infection was not observed in a kinetic analysis of a parallel infection with a single-round YU-2 Env pseudotyped HIV-1_NL4-3 _E^- ^GFP reporter virus (Fig. 8A). Together, these results formally exclude the possibility that the observed increase in the percentage of GFP-positive cells merely reflects a preferential accumulation of cells that were hit during the first round of HIV-1_R7/3 _YU-2 Env GFP infection. In addition, T-cell cultures from n-tg rats challenged with HIV-1_R7/3 _YU-2 GFP never contained GFP-expressing cells above background (data not shown).

Parallel infection with a second replication-competent R5 HIV-1 GFP reporter virus, HIV-1_NL4-3 _92TH014-2 Env GFP, resulted in a productive infection of human T-cells as seen both by an increase in the percentage of GFP-positive cells and p24 antigen in supernatants (Fig. 9A, 9B, right panels). In contrast, neither virological readout provided evidence for a spreading infection in hCD4/hCCR5-tg rat T-cells (Fig. 9A, B, left panels; and data not shown) indicating that the infection phenotype observed for HIV-1_R7/3 _YU-2 Env GFP is not a universal property of replication-competent HIV-1 GFP reporter viruses. Collectively, our results provide evidence that primary rodent T-cells expressing a functional HIV receptor complex have the capacity to support a spreading, most likely cell-to-cell-mediated infection of at least one HIV-1 strain, despite an absence of detectable capsid antigen in culture supernatants.

### Transgenic expression of hCycT1 significantly enhances replication of HIV-1_R7/3 _YU-2 Env GFP

**Figure 10:**
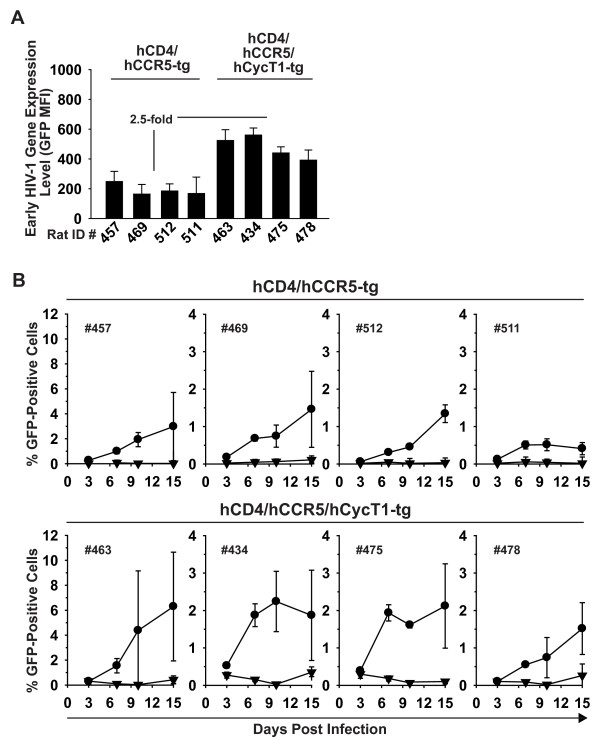
**hCycT1 transgenesis boosts replication of HIV-1_R7/3 _YU-2 Env GFP in T-cells from multi-tg rats**. Activated T-cells derived from the spleens of four double- or four triple-tg rats were challenged with (A) single-round VSV-G HIV-1_NL4-3 _GFP reporter virus and the MFI (GFP) of viable cells was quantified on day 3 p.i. (B) In parallel, T-cell cultures were challenged with HIV-1_R7/3 _YU-2 Env GFP either in the absence (circles) or presence (triangles) of efavirenz (5 μM). Cultures were washed the following day and cultivated for 14 days. At the indicated time points p.i., the percentage of GFP-positive cells was determined by flow cytometry. Shown are the arithmetic mean ± SD of triplicates. In the group of hCD4/hCCR5/hCycT1-tg rats significantly higher replication levels were reached compared to the group of hCD4/hCCR5-tg rats (p = 0.02; linear model variance analysis).

Building on this novel finding, we tested whether transgenic co-expression of hCycT1 affects the replication of HIV-1_R7/3 _YU-2 Env GFP in T-cells from rats expressing the HIV receptor complex. In a first step, we screened a pool of 17 hCD4/hCCR5/hCycT1-tg rats to identify animals with a medium to high level transcriptional responder phenotype. This was achieved by establishing cultures of Ficoll-purified PBMC derived from jugular blood draws of all rats and testing activated T-cell cultures for their ability to support early gene expression following VSV-G HIV-1_NL4-3 _GFP challenge. Cultures from four double-tg rats (rat ID 457, 469, 511, 512) served as reference controls (Fig. 10A). Based on these analyses, four triple-tg rats with a mean enhancement of early HIV-1 gene expression of 2.5-fold were selected (rat ID 434, 463, 475, 478) (Fig. 10A). Subsequently, all eight animals were sacrificed and splenocyte cultures activated with Con A/IL-2 for five days prior to HIV-1_R7/3 _YU-2 Env GFP infection.

All rat T-cell cultures supported an efavirenz-sensitive, spreading infection reflected by an increase of the percentage of GFP-expressing cells over the course of 15 days (Fig. 10B). Of note, individual wells of T-cells from triple-tg rat 463 reached remarkably high percentages of GFP-positive cells (up to 12%) at day 10 and/or 15 p.i. Importantly, cultures from the group of hCD4/hCCR5/hCycT1-tg rats (Fig. 10B, lower panels) displayed a moderate, but significant enhancement of infection compared to the group of hCD4/hCCR5-tg animals (Fig. 10B, upper panels) (p = 0.02; linear model variance analysis). Finally, we assessed whether the infection of HIV-1_R7/3 _YU-2 Env GFP or of prototypic R5 HIV-1 wildtype viruses in triple-tg rat T-cell cultures can be detected by quantification of cell-associated levels of p24 antigen over time. While this was possible for infected human T-cell cultures with levels ranging from 1-11 ng/ml, no p24 levels above background were found in rat T-cells (data not shown). Collectively, based on the kinetics and characteristics of cell-associated early gene expression, hCycT1 transgenesis can facilitate replication of an HIV-1 GFP reporter virus in primary rat T-cells.

## Discussion

To further develop a small animal model that is highly permissive for *de novo *infection by HIV-1, we sought to expand on our earlier results in hCD4/hCCR5-tg rats [3,7,12,13]. Here, we generated rats that transgenically express hCycT1 in a cell type-specific manner to explore their suitability for enhancing the susceptibility of this important cell type in the multi-tg rat model.

In infected rat T-cells, hCycT1 transgene expression boosted early HIV gene expression ~3- to 7-fold compared to n-tg littermates. Importantly, cultures from ~1/4 of hCycT1-tg rats reached levels of HIV gene expression within the average range of infected human T-cells. However, while hCycT1 expression was clearly required, no correlation could be established between steady-state expression levels of hCycT1 and the ability of infected cultures to support early HIV gene expression. This finding is consistent with a scenario in which endogenous factors in concert with hCycT1 play a decisive role in regulating HIV LTR-driven gene expression in rat T-cells. Alternatively, only a subset of CD4 T-cells may be capable of translating hCycT1 expression into an enhanced gene expression and in these cells transgene expression levels may still correlate with the functional impact.

In principle, this could involve the recruitment of transcription factors (e.g., NF-κB, SP1, NFAT) to the 5'-LTR as well as factors regulating the activity of P-TEFb. Interestingly, no cellular gene is as sensitive to the availability of P-TEFb as the genes of HIV-1 (for review [17]). So far, several positive regulators of P-TEFb and HIV-1 LTR transactivation have been reported, including bromodomain protein Brd4 [29,30], NF-κB [31], and the DNA-dependent ATPase subunit Brm of the SWI/SNF chromatin-remodeling complex [32]. Negative regulators have also been found, including the noncoding 7SK small nuclear RNA [33,34], HEXIM1 [35], the DRB sensitivity-inducing factor (DSIF) [36], and negative elongation factor (NELF) [37]. Furthermore, transcription factor recruitment at contiguous LTR regions is partly dependent on histone acetylation as well as the viral Tat protein [38]. Conceivably, the interaction of such endogenous factors with the Tat/hCycT1-containing P-TEFb complex may be different in rats than in humans. Similarly, dominant-negative activities of CycT1 or CycT2 of rat origin have to be considered in a hCycT1-tg context.

Recently, Sun *et al. *reported that hCycT1 transgenesis in mice, employing the identical transgene vector, also resulted in a cell type-specific expression in CD4 T-cells, macrophages as well as microglia [25,26]. Importantly, crossing HIV-1_JR-CSF_-tg mice, which carry two to four proviruses, with these hCycT1-tg mice revealed a marked increase in the production of infectious HIV-1 in both T-cells and macrophages [25]. HIV-1 p24 concentrations in supernatants were still lower than the levels typically reached in dynamically infected human cultures. In a more refined characterization of T-cells from these hCycT1-tg mice, Zhang *et al. *demonstrated HIV-1 RNA expression per infected T-cell was only 10% of that of human references [10], indicating that the transcriptional deficit also in this rodent had only partially been overcome by hCycT1 transgenesis. Of note, this analysis required a secondary T-cell receptor stimulation to circumvent a peri-integrational block in primary mouse T-cells, which is absent in the rat species [3]. Interestingly, infected rat macrophages pose an exception to the otherwise species-specific impairment at the level of early HIV-1 gene expression [3]. As shown herein, tg expression of hCycT1 does not translate into an enhanced viral gene expression in this primary cell type, and already macrophages from n-tg rats are at a level comparable to human MDM. This may, in part, relate to the ability of HIV-1 to exploit a distinct set of nuclear transcription factors and alternative mechanisms of transcriptional regulation in macrophages compared to other cell types, including T-cells (for review [39-41]). In addition, hCycT1-dependence and species-specific differences of HIV LTR driven gene expression appear to be less pronounced or even absent when levels of gene expression per cell are low. This was observed both for macrophages (Fig. 5C) and T-cells infected with the HIV-1 strain R7/3 with apparently lower intrinsic LTR activity (Fig. 8C, compare left and right panel). Notably, macrophages from hCD4/hCCR5-tg rats are the only primary non-human cells reported to allow a productive HIV-1 infection, albeit at lower levels than in human MDM [4,12,42]. The general ability of macrophages to support HIV LTR-driven transcription and, related to that, their responsiveness to transgenic hCycT1 expression, appears to be remarkably different in rats and mice [25].

Zhang *et al. *also characterized the late phase in primary T-cells from hCycT1-tg mice *ex vivo *and report post-transcriptional defects at the levels of Gag expression, Gag processing, Gag release and virus infectivity [10]. They estimated that the post-integration defects alone add up to a 300- to 500-fold reduction in the yield of infectious virus after a single cycle of HIV-1 replication. It is currently unclear why T-cells from provirus-tg mice appear to be much less restricted in supporting these steps of the HIV-1 replication cycle [24-26]. Limitations for several distinct late-phase steps in rodent cells have been proposed, including the Rev-dependent export of HIV-1 RNA [1,9,43-45] Gag trafficking and virion assembly [1,9,10,45], both inhibitory and supportive functions of TRIM family members [46], as well as the Vif-resistance of mouse APOBEC3G [47]. These findings are of high relevance for future analyses of the late-phase defect in T-cells from rats and possible additional genetic modifications of the host.

We identify HIV-1_R7/3 _YU-2 Env GFP as a virus, the behavior of which in tg rat T-cells *ex vivo *displays key characteristics of a spreading, primarily cell-to-cell-mediated infection: first, GFP expression from the *nef *locus, a surrogate for early viral gene expression in infected cells, increased continuously over periods of 2 weeks with peak levels comparable to human reference cultures. This was not a general property of replication-competent HIV-1 GFP reporter viruses since another R5 strain failed to recapitulate this phenotype. Second, this increase was sensitive to efavirenz addition 18 h p.i. and also not seen for an *env*-deficient single-round virus. This demonstrates the requirement for multiple rounds of replication involving reverse transcription. Third, the lack of significant p24 levels in supernatants from infected rat T-cell cultures and the successful transfer of infection to naïve cultures only through cells, but not supernatant from infected cultures, indicates a primarily cell-to-cell-mediated spread. Levels of p24 antigen per infected rat T-cell as well as the overall percentage of infected T-cells in culture may have been too low to detect a spread by quantification of cell-associated p24 levels. This challenging observation warrants further investigations. It will be interesting to investigate the genetic determinants in HIV-1_R7/3 _YU-2 Env GFP that underlie its ability to propagate in tg rat T-cells. Through such a genetic approach and forced adaptation of this or other HIV-1 strains for replication in the improved transcriptional context of triple-tg rat T-cells, the evolution of a highly rat-adapted HIV-1 strain may be feasible. Moreover, the molecular characterization of such an adapted strain could greatly facilitate the identification of host determinants that are critical regulators of late phase-steps of HIV replication.

## Methods

### Animals

The hCD4/hCCR5-tg rats have been reported [12]. The tg vector pMΦE4A.CyclinT1 (Fig. 1A), which encodes a 1.15-kb cDNA fragment of hCycT1 and carries a cassette containing the murine CD4 enhancer/promoter and the human CD4 intronic sequence, which encompasses the macrophage enhancer and the CD4 silencer elements was recently described [25]. For generation of hCycT1-tg rats, the transgene vector was linearized using the flanking *Not*I sites and microinjected into male pronuclei of fertilized oocytes from outbred Sprague-Dawley rats. Founders for hCycT1-tg rats were identified by PCR amplification of a hCycT1-specific sequence in tail biopsy DNA samples (5'-primer: GAT ACT AGA AGT GAG GCT TAT TTG, 3'-primer: CAG ATA GTC ACT ATA AGG ACG AAC) and selected for further matings with n-tg Sprague-Dawley rats. Transgene expression in progeny was demonstrated by western blot detection of hCycT1 in thymocyte extracts. Heterozygous hCD4/hCCR5/hCycT1-tg animals were generated by interbreeding of homozygous hCD4/hCCR5-tg rats with hCycT1-tg rats. Animals were kept in the IBF animal facility of Heidelberg University.

### Cells

The set-up and cultivation of primary cultures for T-cells and macrophages isolated from buffy coats of human donors or rat spleen have been described [4,12]. PBMC from rat blood were purified and cultured by drawing 1.5 ml of peripheral blood from the *Vena jugularis *of anaesthetized rats and isolating the mononuclear cells on Ficoll-hypaque gradients. PBMC were activated using recombinant human interleukin-2 (IL-2) (20 nM; Biomol) in combination with phytohemagglutinin-P (human) or concanavalin A (Con A) (rat) (each at 1 μg/ml, Sigma). At the time of HIV-1 infection cultures typically contained ~90–95% CD3-positive T-cells, with NK cells constituting the majority of non-T-cells [27,42]. The original sources and cultivation of TZM-bl and 293 T cells has been reported [4].

### Viruses

The molecular clone pNL4-3 E^-^-EGFP, encoding an *env*-defective replication-deficient HIV-1_NL4-3 _carrying an *egfp *gene within the *nef *locus (HIV-1_NL4-3 _GFP) [48], was a gift of Nathaniel Landau via the NIH AIDS Research and Reference Program. The HIV-2 molecular clone pROD-A, encoding an *env*-defective replication-deficient HIV-2-based virus carrying an *egfp *gene within the *nef *locus (HIV-2_ROD-A _GFP) was provided by Matthias Dittmar [49]. The production of VSV-G pseudotyped HIV stocks has been described [4]. The replication-competent R5 strains HIV-1_R7/3 _YU-2 Env GFP [28] and HIV-1_NL4-3 _92TH014-2 Env GFP [50] were gifts from Mark Muesing and Jan Münch, respectively. The first strain has a truncated *vpr *gene and is devoid of a functional *vpu *gene [28]. The original sources of HIV-1_YU-2_, HIV-1_Ba-L_, and HIV-1_JR-FL _have been reported [12]. Virus-containing supernatants were concentrated using Centricon Plus-70 spin columns (Millipore) and then purified through a 20% sucrose cushion (27,000 g, 4°C, 60 min). The virion-enriched pellet was resuspended in culture medium, frozen in liquid nitrogen and stored at -80°C. All HIV stocks were characterized for infectious titer on TZM-bl cells and HIV-1 stocks for p24 concentration by ELISA [42]. For analysis of cell-associated p24 levels, cells were lysed in 1% Triton X-100 in PBS-Tween and quantified by ELISA.

### HIV Tat and LTR reporter constructs

pBC12/CMV HIV-2 Tat [51] (pHIV-2 Tat) was a gift from Brian Cullen. pCMV4-Tat2ex carries the second exon of the *tat *gene from HIV-1_NL4-3 _(pHIV-1 Tat). To generate minimal HIV LTR reporter plasmids, HIV LTR and gag-leader sequences were amplified from pNL4-3 and pROD10, respectively, and subcloned via introduced *Ase*I and *Nhe*I sites in pEGFP-N1 vector (Invitrogen) replacing the CMV-IE promoter.

### Western blot analysis

Cells were lysed in buffer A (50 mM HEPES, 135 mM NaCl, 10% glycerol, 1% Triton X-100, 1 mM EDTA), and 1 × protease inhibitor cocktail (Sigma), pH 7.2, for 1 h at 4°C. Lysates were collected after centrifugation at 13,200 × g for 20 min at 4°C and analyzed for protein concentration using the BCA protein assay (Pierce). Proteins were run on a 12% SDS-PAGE and transferred onto nitrocellulose. Blots were sequentially probed with mouse anti-hCycT1 monoclonal antibody sc-8127 and rabbit antiserum sc-153 against ERK2 (both from Santa Cruz). After secondary antibody treatment, the blots were exposed to autoradiographic films using the ECL system.

### Antibody-coupled bead selection of rat splenocytes

rCD4- and rCD8-positive primary cells were purified from freshly isolated rat splenocytes by positive selection with the MACS bead technology (Miltenyi Biotec). As primary reagent, phycoerythrin (PE)-conjugated monoclonal antibodies against rCD4 (clone OX-8) or rCD8 (clone OX-35; both from BD Pharmingen) were used followed by anti-PE magnetic beads. The column purification of labeled cells was carried out according to the manufacturer's instructions, and the purity of selected cell populations was analyzed by flow cytometry.

### Nucleofection

Activated primary rat T-cells were transfected with proviral reporter plasmids, or with LTR and Tat plasmids, by nucleofection and analyzed by flow cytometry one day later, in principle as reported [27].

### Flow cytometry

Cells were either nucleofected with pHIV-1_NL4-3 _GFP, pHIV-2_ROD-A _GFP, or pHIV-1_NL4-3 _LTR GFP, pHIV-2_ROD-A _LTR GFP in the absence or presence of Tat expression plasmids, or infected with the corresponding VSV-G pseudotypes, or HIV-1_R7/3 _YU-2 Env GFP, or HIV-1_NL4-3 _92TH014-2 Env GFP. At the indicated time points viable cells were analyzed for the MFI of GFP in and/or the percentage of GFP-positive cells on a FACSCalibur using BD CellQuest Pro 4.0.2 Software (BD Pharmingen).

### Statistics

Unpaired Student's *t*-test analysis was calculated with the software MS Excel 2003. For the data presented in Fig. 10B a linear model variance analysis (repeated measurement design) was performed. A result was considered significant when p < 0.05.

## Competing interests

The authors declare that they have no competing interests.

## Authors' contributions

NM, CG, WCG, MAG and OTK designed the study and interpreted the data. KG and IA provided technical support. VNKR and DRL provided the pMΦE4A.CyclinT1 tg vector and gave input on the manuscript. MS gave input on data interpretation and on the manuscript. NM, CG and OTK wrote the paper.
